# A Novel Technique for Transvaginal Retrieval of Enlarged Pelvic Viscera during Minimally Invasive Surgery

**DOI:** 10.1155/2012/454120

**Published:** 2012-06-28

**Authors:** Allison Wyman, Lauren Fuhrig, Mohamed A. Bedaiwy, Robert DeBernardo, Gary Coffey

**Affiliations:** Divsion of Gynecologic Oncology, Department of Obstetrics and Gynecology, Seidman Cancer Center, University Hospitals Case Medical Center, School of Medicine, Case Western Reserve University, 11000 Euclid Avenue, Cleveland, OH 44106, USA

## Abstract

*Introduction*. With the widespread adoption of laparoscopic and robotic surgery, more and more women are undergoing minimally invasive surgery for complex gynecological procedures. The rate-limiting step is often the delivery of an intact uterus or an unruptured adnexal mass. To avoid conversion to a minilaparotomy for specimen retrieval, we describe a novel technique using an Anchor Tissue Retrieval System bag in conjunction with a pneumo-occluder to easily retrieve large specimens through a colpotomy incision. *Surgical Technique*. After completion of the robotic-assisted hysterectomy, the uterus, fallopian tubes, and ovaries were too large to be retrieved intact despite multiple attempts of delivery through the colpotomy incision. Prior to resorting to a minilaparotomy or morcellation of the specimen, a 15 mm anchor retrieval bag with a pneumo-occluder was placed through the vagina and the intact specimen was easily placed inside the bag under direct visualization and removed through the colpotomy incision intact. *Conclusion*. We routinely utilize this technique to retrieve hysterectomy specimens that are not readily delivered through the colpotomy incision and find this technique to be safe, highly efficient, and cost effective when there is a need to remove large intact specimens during minimally invasive surgery.

## 1. Introduction 

Minimally invasive surgeries for gynecological conditions are becoming more common, especially since the wide-spread adoption of robotic surgery. As surgeons grow increasingly comfortable with complex laparoscopic and robotic procedures, the rate-limiting step often becomes specimen retrieval through a small incision. In circumstances where malignancy is not a concern, specimen retrieval can be challenging, often resorting to morcellation of the specimen and at times enlarging a port site to facilitate removal of the specimen. These practices increase the likelihood of contamination, implantation at the port site, port site hernias, tissue trauma, and increased operative time. 

Delivery of an intact specimen through the colpotomy incision presents its own unique challenges. While the colpotomy incision is larger than a typical 10 mm port site, delivering a specimen through this vaginal incision is often difficult, time consuming, and can result into trauma to nearby structures. Improperly grasping the specimen can result in inadvertently incorporating nearby organs, such as the bowel or rectosigmoid epiploica. Inadequately grasping the specimen often results in tearing or laceration of the specimen during each attempt at delivery through the colpotomy incision. This problem is compounded in virginal, nulliparous, and morbidly obese patients. 

The importance of this matter is highlighted in women having surgery for gynecologic malignancy where an intact specimen is required for histopathological staging. The issue is most commonly encountered in women having surgery for endometrial cancer. Intact removal of the specimen is essential to preserve the architectural features. Interpretation of tumor depth of invasion and lymphovascular spread are important prognostic factors. The benefits of minimally invasive surgery may not outweigh the risks of compromising the ability to adequately interpret the pathologic specimen. For example, at our institution, patients diagnosed on final pathology with endometrial cancer after morcellation are offered pelvic radiation therapy that otherwise could have been avoided if delivery of an intact uterus and specimen was successful at the time osf the procedure. 

The objective of this paper is to describe a novel technique to facilitate intact retrieval of large specimens during a minimally invasive hysterectomy. 

## 2. Surgical Technique 

A robotic-assisted hysterectomy and bilateral salpingooophorectomy were performed for a patient with a preoperative diagnosis of atypical endometrial hyperplasia. The operation was uncomplicated until attempts were made to deliver the intact specimen through the vagina. Traction on the uterine manipulator during attempted delivery resulted in the specimen falling off the uterine manipulator. A ringed forceps and then a single-tooth tenaculum were both used in attempt to retrieve the specimen. Despite multiple attempts, none of the efforts were successful, and the specimen was lacerated in the process. 

**Figure 1:**
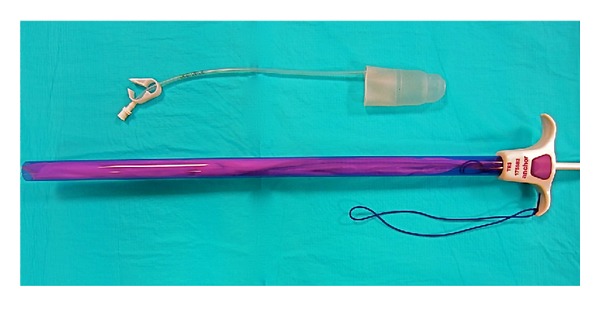
Pneumo-occluder and 15 mm anchor device.

**Figure 2:**
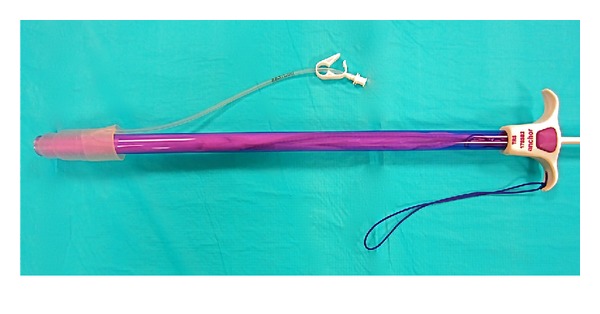
Pneumo-occluder placed onto tip of device.

**Figure 3:**
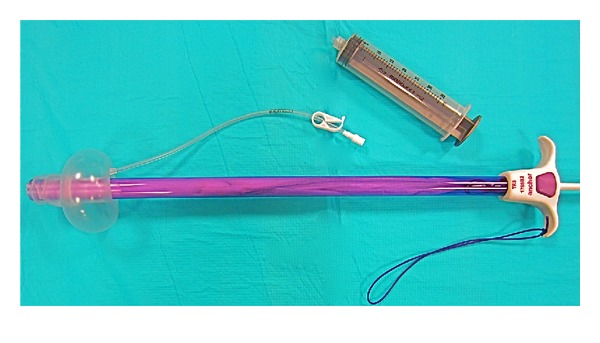
60 cc syringe used to expand pneumo-occluder.

**Figure 4:**
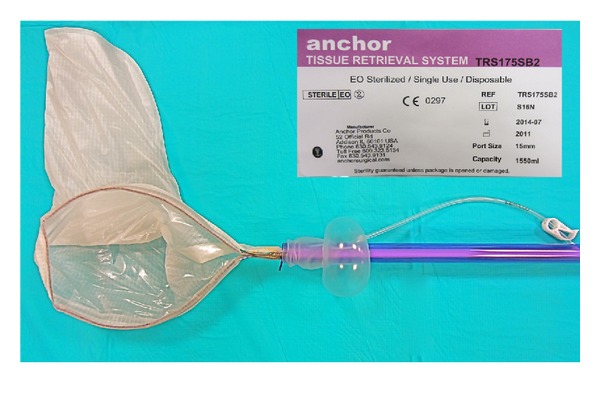
Anchor bag expelled to obtain specimen.

In a final attempt prior to undocking the robot and converting to a minilaparotomy, the specimen was grasped by the robotic arm and elevated off the pelvic floor. A 15 mm Anchor Tissue Retrieval System no. TR190SB2 retrievable bag along with a standard KOH Colpotomizer System pneumo-occluder balloon was used in a novel approach for specimen retrieval through the colpotomy incision (Figures 1 and 2). First to ensure adequate pneumoperitoneum, the donut-shaped pneumo-occluding balloon was placed just over the shaft of the retrieval bag and inflated (Figure 3). The complete apparatus was then inserted into the vagina under direct visualization, and the pneumo-occluder balloon was inflated. After a pneumoperitoneum was obtained, excellent visualization was noted. The bag was deployed (Figure 4) and the uterus, fallopian tubes, and ovaries were easily placed inside the bag without difficulty using the robotic arm. The bag was then closed, and the entire apparatus was effortlessly removed through the vagina with the complete specimen safely encapsulated and preserved. The final pathology demonstrated FIGO stage I endometrial adenocarcinoma with mucinous features with 12% myometrial invasion and no LVI. 

Placement of the specimen within the retrieval bag is straightforward and easy to adapt to any minimally invasive gynecological surgery. After completion of the colpotomy incision, the uterus is grasped and elevated. The assistant assembles the apparatus, as illustrated in Figure 2. A KOH Colpotomizer System pneumo-occluder is slipped onto a 15 mm anchor retrieval system bag and inserted into the vagina. After inflating the pneumo-occluder, a pneumoperitoneum is reestablished which is essential for adequate visualization. 

**Figure 5:**
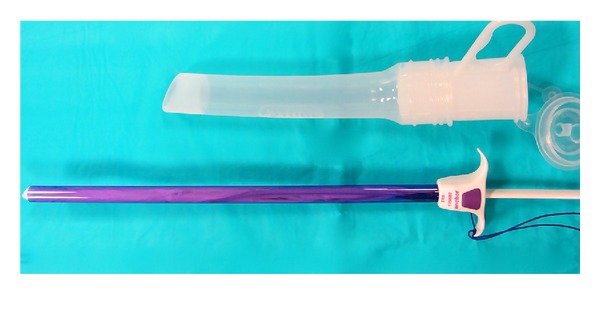
McCartney tube as an alternative to pneumo-occluder.

**Figure 6:**
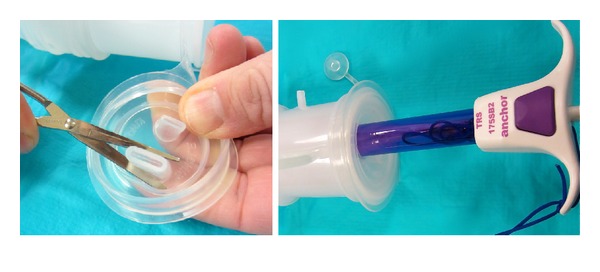
Cut tip to insert anchor bag device within the McCartney Tube.

**Figure 7:**
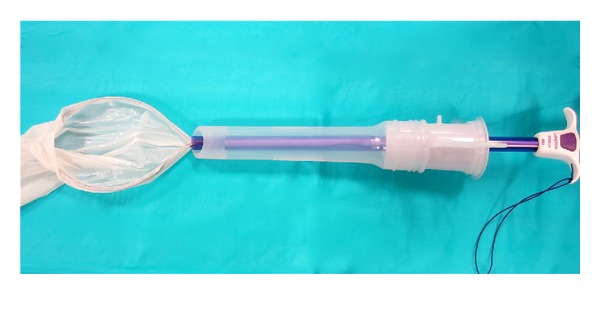
Apparatus assembled for specimen retrieval.

Alternatively, for surgeons that use a McCartney tube (Gate Healthcare) rather that the KOH for TLH or robotic hysterectomy, the device can be easily modified as illustrated in Figures 5, 6, and 7 to accomplish the same ends. This is also demonstrated in Video Clip 1 A (see the Supplementary Material available online at doi:10.115/2012/454120) for removal of hysterectomy specimen and in Video Clip 2 A for removal of pelvic lymph node dissection. 

Without the use of the McCartney tube, the retrieval system bag apparatus is introduced through the colpotomy incision and deployed. The specimen is placed in the bag and it is closed. Once the specimen is secured, the vaginal assistant applies downward traction onto the apparatus to extract the mass through the vaginal canal. The force applied to the specimen within the bag squeezes the viscera smaller facilitating its delivery while simultaneously maintaining its architectural integrity. Furthermore, the specimen is removed without spillage or contamination. 

We have successfully retrieved large and challenging specimens that could barely fit in the 15 mm Anchor Tissue Retrieval System (no. TR190SB2). The bag has a total volume capacity of 1860 mL accommodating very large specimens. In addition, lubrication can be applied to the vagina or the outside of the bag without compromising the surgeon's grip on the specimen. Our experience suggests that the anchor bag is superior to the EndoCatch bag, as more force can be exerted upon the anchor product prior to bag failure. In addition, the anchor retrieval system can be used multiple times during a case. 

## 3. Discussion 

In this paper we described a simple yet novel approach for specimen retrieval that promises to decrease operative time by facilitating safe and intact removal of large specimens following complex surgical procedures by minimally invasive approaches. The technique itself can be easily adopted and mastered by any minimally invasive surgeon. There is no additional cost associated with this technique, provided the surgeon was planning on using a retrieval system such as EndoCatch or the anchor product. However, a prospective study would be imperative to compare the actual decrease in operative time, conversion to mini-laparotomy, and operative expenses associated with this new technique. 

Minimally invasive surgeries for gynecologic conditions are becoming more common due to the technical advantages of robotic surgery and the increasing comfort level and experience of advanced laparoscopic surgeons. As increasing complex procedures becomes a more commonplace for gynecological surgeons, technological advancements will need to be made to overcome new challenges facing minimally invasive surgeons. Facilitating retrieval of specimens, especially large or cancer-bearing organs, during minimally invasive surgery is paramount for the success of a minimally invasive procedure. This is most apparent for women undergoing surgery for endometrial cancer; however, this retrieval technique may have applications for even a wider range of minimally invasive surgical procedures. At our institution, we commonly use this technique to remove lymphatic tissue and large adnexal masses. This technique has also been used to remove an intact kidney and a segment of large intestine. 

Feasibility and safety of laparoscopic and robotic hysterectomies have been demonstrated in several studies [1–4]. The benefits of this approach are readily apparent: more rapid recovery, shorter hospital stay, and less pain than conventional surgery [1, 2]. While these advantages are important, a minimally invasive approach is not warranted if it compromises the oncologic outcome. This is best demonstrated in patients with endometrial cancer. In these cases, adjuvant therapy is dictated by histologic grade, depth of myometrial invasion, and lymphovascular space invasion. Morcellating or fragmenting a hysterectomy specimen during retrieval not only limits the pathologic evaluation but it can also lead to seeding the abdominal and pelvic peritoneum [4]. In cases where malignancy is not a primary concern, alternative methods of retrieval when the uterine manipulator become dislodged such as using a tenaculum or ring forceps have been described [5]. Although occurrences are rare, aggressive attempts to deliver a difficult specimen through the colpotomy incision can lead to unintended injury to the rectum or small bowel [5]. Lastly, surgeons that perform minimally invasive hysterectomies on a routine basis know that precious time is wasted with fruitless attempts to deliver a uterus that is too large to fit through a small and narrow vagina as the case above demonstrates. 

Since the routine adoption of this technique at our institution, we have successfully used the technique in approximately 100 cases and have found specimen retrieval is less time consuming and less frustrating during minimally invasive hysterectomy. In addition, the incidence of conversion to mini-laparotomy for specimen retrieval has been impacted. Since adoption of this technique, there has not been one instance where conversion was preformed solely for specimen retrieval. Data to support our observations are difficult to quantify as the time required to remove a specimen after completion of the vaginal colpotomy has not been routinely recorded at our institution. Nonetheless, over the last 30 cases preformed by one author, the average time to retrieve specimens that could not be spontaneously removed with the uterine manipulator was less than 2 minutes, ranging from 44 seconds to 3 minutes and 25 seconds.

Since the introduction of this novel technique, we have found less time is required to remove large specimens. Total operative time is shorter which, in theory, can lead to a decrease of overall cost of robotic hysterectomy. Despite numerous publications on the cost effectiveness of laparoscopic and robotic surgery, there is an equally valid argument that, in terms of dollars spent per case, conventional surgery is considerably less expensive. This issue will become more important as healthcare reimbursement becomes increasingly limited. Multiple papers have addressed the higher cost for robotic hysterectomy and conventional laparoscopic hysterectomy [2, 3]. Any new surgical technique that is cost effective and has the potential to decrease the overall cost of these procedures warrants further investigation. 

In conclusion, the technique described above is a simple adjunct to aid retrieval of large uteri and masses through a colpotomy incision. We now use this technique almost exclusively when operating on women with endometrial cancer when the uterus does not deliver spontaneously with the uterine manipulator in an attempt to minimize exposure of cancer-bearing tissue to the pelvis.

## Supplementary Material

Supplementary Material: includes three video clips (1, 2, and 3) demonstrating the novel surgical technique during minimally invasive surgery. Video clip 1 is a routine laparoscopic hysterectomy and bilateral salpingoopherectomy with removal of the pelvic viscera using the retrieval system. Video clip 2 and 3 demonstrate the removal of a hysterectomy specimen and removal of pelvic lymph node dissection using the modified McCartney technique.
